# Biochanin A Mitigates Atherosclerosis by Inhibiting Lipid Accumulation and Inflammatory Response

**DOI:** 10.1155/2020/8965047

**Published:** 2020-11-11

**Authors:** Xiao-Hua Yu, Jiao-Jiao Chen, Wen-Yi Deng, Xiao-Dan Xu, Qi-Xian Liu, Meng-Wen Shi, Kun Ren

**Affiliations:** ^1^Institute of Clinical Medicine, The Second Affiliated Hospital of Hainan Medical University, Haikou, 570100 Hainan, China; ^2^Department of Pathology, The First Affiliated Hospital of Anhui Medical University, Hefei, 230032 Anhui, China; ^3^The First School of Clinical Medicine, Anhui Medical University, Hefei, 230032 Anhui, China; ^4^Department of Pathophysiology, School of Basic Medical Sciences, Anhui Medical University, Hefei, 230032 Anhui, China

## Abstract

Biochanin A (BCA), a dietary isoflavone extracted from red clover and cabbage, has been shown to antagonize hypertension and myocardial ischemia/reperfusion injury. However, very little is known about its role in atherogenesis. The aim of this study was to observe the effects of BCA on atherosclerosis and explore the underlying mechanisms. Our results showed that administration of BCA promoted reverse cholesterol transport (RCT), improved plasma lipid profile, and decreased serum proinflammatory cytokine levels and atherosclerotic lesion area in apoE^−/−^ mice fed a Western diet. In THP-1 macrophage-derived foam cells, treatment with BCA upregulated ATP-binding cassette (ABC) transporter A1 (ABCA1) and ABCG1 expression and facilitated subsequent cholesterol efflux and diminished intracellular cholesterol contents by activating the peroxisome proliferator-activated receptor *γ* (PPAR*γ*)/liver X receptor *α* (LXR*α*) and PPAR*γ*/heme oxygenase 1 (HO-1) pathways. BCA also activated these two signaling pathways to inhibit the secretion of proinflammatory cytokines. Taken together, these findings suggest that BCA is protective against atherosclerosis by inhibiting lipid accumulation and inflammatory response through the PPAR*γ*/LXR*α* and PPAR*γ*/HO-1 pathways. BCA may be an attractive drug for the prevention and treatment of atherosclerotic cardiovascular disease.

## 1. Introduction

Cardiovascular disease (CVD) remains the leading cause of mortality and disability worldwide. Atherosclerosis is the pathological basis of most CVD, such as unstable angina, myocardial infarction, and stroke [1]. This is a complex pathological process characterized by lipid deposition and chronic inflammation within the arterial wall [2]. During atherogenesis, circulating monocytes adhere to endothelium, migrate into the subendothelial space, and then differentiate into macrophages. After uptake of oxidized low-density lipoprotein (ox-LDL), macrophages are transformed into foam cells, a hallmark of early-stage atherosclerotic plaques [3]. On the other hand, ox-LDL can stimulate macrophages to produce multiple proinflammatory factors, further promoting lipid accumulation and aggravating plaque formation [4]. Despite statin monotherapy or its combination with other drugs such as evolocumab has obtained considerable improvement in clinical outcomes of CVD patients, the residual risk is still a major challenge [5, 6]. Thus, there is an urgent need to develop new atheroprotective drugs for decreasing the risk of CVD.

Reverse cholesterol transport (RCT) is a process whereby excessive cholesterol in peripheral tissues and cells is transported by high-density lipoprotein (HDL) to the liver for excretion into feces. ATP-binding cassette (ABC) transporters A1 (ABCA1) and ABCG1, two membrane proteins, play a central role in mediating intracellular cholesterol efflux, the first and rate-limiting step of RCT [7, 8]. Previous studies from our laboratory and other groups have demonstrated that fargesin, polydatin, and N-acetylneuraminic acid promote RCT and diminish atherosclerotic lesion area in apolipoprotein E-deficient (apoE^−/−^) mice by upregulating ABCA1 and ABCG1 expression [9–11]. Peroxisome proliferator-activated receptor *γ* (PPAR*γ*) and liver X receptor *α* (LXR*α*) belong to nuclear receptors [12]. Heme oxygenase (HO-1) is a critical enzyme in the heme catabolism. It has been reported that activation of LXR*α* and HO-1 not only increases ABCA1 and ABCG1 expression but also inhibits proinflammatory cytokine secretion [13–15]. Interestingly, PPAR*γ* serves as an upstream effector of LXR*α* and HO-1 [12, 16, 17]. These findings suggest that PPAR*γ* functions as a crosstalk between lipid metabolism and inflammation. Activation of PPAR*γ* is a promising strategy for the prevention and treatment of atherosclerosis.

**Figure 1:**
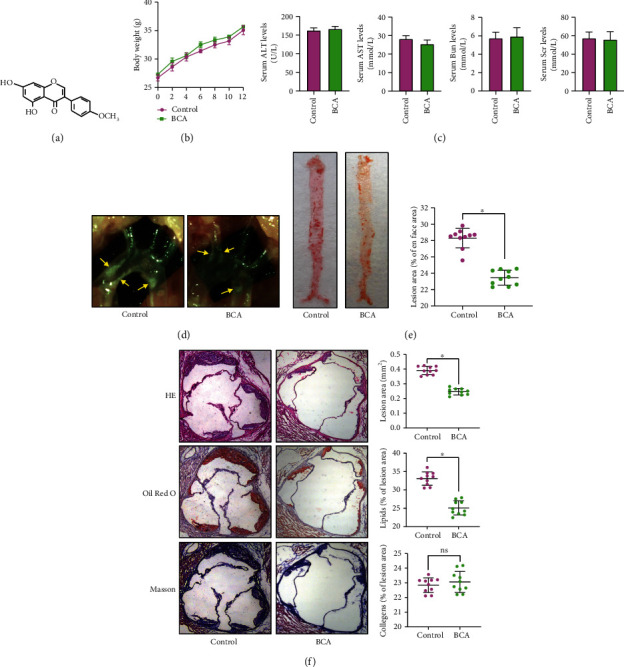
BCA ameliorates atherosclerosis in apoE^−/−^ mice. Forty male apoE^−/−^ mice were administered with BCA (50 mg/kg) or vehicle by oral gavage and fed a Western diet for 12 weeks (*n* = 20 per group). (a) Chemical structure of BCA. (b) Comparison of body weight. (c) Detection of serum levels of ALT, AST, Bun, and Scr. (d) The plaques (yellow arrows) in the aortic arch of apoE^−/−^ mice under a stereoscopic microscope. (e) The atherosclerotic lesion area of the whole aorta was analyzed by Oil Red O staining (*n* = 10 per group). (f) Sections of the aortic root were stained with HE, Oil Red O, or Masson. The percentage of lesion area, lipids, and collagens were quantified (*n* = 10 per group) using Image-Pro Plus 7.0 software. Data are expressed as mean ± SD. ^∗^*P* < 0.05 vs. control group.

Biochanin A (BCA, Figure 1(a)), also known as 5,7-dihydroxy-4′-methoxy-isoflavone, is an isoflavone present in red clover, cabbage, alfalfa, and many other herbal products [18]. It is suggested that BCA exerts multiple pharmacological effects, such as antioxidation, anti-inflammation, and anti-infection [19, 20]. Recent studies showed that BCA is protective against obesity, hypertension, and myocardial ischemia/reperfusion injury [21, 22]. However, it is still unclear whether BCA is able to inhibit the development of atherosclerosis.

In this study, we found that BCA reduces atherosclerotic lesion area in apoE^−/−^ mice. Mechanistically, BCA activates the PPAR*γ*/LXR*α* and PPAR*γ*/HO-1 pathways to upregulate ABCA1 and ABCG1 expression, leading to increased macrophage cholesterol efflux and RCT. On the other hand, BCA inhibits proinflammatory cytokine secretion by activating these two pathways.

## 2. Materials and Methods

### 2.1. Mice and Diet

Forty male 8-week-old apoE^−/−^ mice on a C57BL/6 background were purchased from Changzhou Cavens Lab Animal Co., Ltd (Jiangsu, China) and housed under a 12 h light/dark cycle in an environmentally controlled room (24 ± 2°C, 60% humidity). Mice were fed a Western diet containing 21% fat and 0.3% cholesterol and randomized into two groups (*n* = 20 per group): control group and BCA group. Mice in BCA group were intragastrically administered with 50 mg/kg BCA (Sigma-Aldrich, St. Louis, MO, USA) dissolved in 1% dimethyl sulfoxide (DMSO) twice every week, and control mice were treated with an equal volume of vehicle. Body weight of each mouse was recorded once every two weeks. Twelve weeks later, all mice were sacrificed, and the heart, aorta, and blood were collected. Sodium pentobarbital anesthesia was conducted throughout all surgeries. Prior to euthanasia, 4% thioglycollate broth was intraperitoneally injected into mice, followed by injection with 5 mL of sterile PBS for the collection of mouse peritoneal macrophages (MPMs). After centrifugation (300 rpm, 5 min, 4°C), the isolated MPMs were counted and then cultured in RPMI 1640 medium (Gibco, Grand Island, NE, USA) containing 10% fetal bovine serum (FBS, Gibco). All experiments were conducted according to protocols approved by the Animal Care and Use Committee at the Second Affiliated Hospital of Hainan Medical University.

### 2.2. Measurement of Serum Biochemical Indicators

The blood samples were obtained from the retro-orbital plexus and then stored at 4°C for 4 h. Followed by centrifugation at 3000 rpm for 10 min, the supernatants were collected as serum samples for further analyses. Serum levels of alanine aminotransferase (ALT), aspartate transaminase (AST), urea nitrogen (BUN), and creatinine (Scr) were quantified using specific kits (Guide Chem, Zhejiang, China).

### 2.3. Evaluation of En Face Lesion Area

After euthanasia, the whole aorta was dissected from apoE^−/−^ mice. The adventitial tissues were carefully removed. The aorta was unfolded longitudinally, stained with Oil Red O, and then photographed. The percentage of lesion area stained by Oil Red O in the aortic surface was determined by Image-Pro Plus 7.0 software.

### 2.4. Assessment of Atherosclerotic Lesions in the Aortic Root

The upper part of the heart and proximal aorta were isolated after euthanasia. Then, the heart was rinsed with PBS, embedded in Optimal Cutting Temperature compound (OCT, Sakura Finetek Japan Co., Ltd, Tokyo, Japan), and serially sectioned (8 *μ*m thick) throughout the three aortic valves. The sections were subjected to hematoxylin-eosin (HE), Oil Red O, and Masson staining to evaluate plaque area, lipid deposition, and collagen content, respectively. Quantitative analyses were conducted by means of Image-Pro Plus 7.0 software.

### 2.5. In Vivo RCT Assay

RCT was detected according to our previous method [23]. J774 macrophages were cotreated with acetylated LDL (50 *μ*g/mL, Yiyuan biotechnology, Guangzhou, China) and [^3^H]-cholesterol (5 *μ*Ci/mL, PerkinElmer, MA, USA) for 48 h. Following equilibration and resuspension in ice-cold Dulbecco's modified Eagle's medium (DMEM, Gibco), the labeled cells were injected into the abdominal cavity of experimental mice (4.5 × 10^8^cells/mouse, *n* = 5 per group). After 6, 24, and 48 h of injection, plasma samples were collected through saphenous vein puncture, 10 *μ*L aliquots of which was used to determine the radioactivity of [^3^H]-cholesterol using a liquid scintillation counter. The feces were continuously gathered from 0 to 48 h, freeze-dried, weighed, and soaked in ethanol. The radioactivity in 20 *μ*L of aliquots was measured. At the end of the study, the liver tissues were isolated from euthanized mice, washed in ice-cold PBS, dried on filter papers, weighed, and stored at -20°C. Then, 100 mg of frozen hepatic tissue was mixed with isopropanol/hexane (2:3, *V*/*V*) for 48 h and then dried overnight for lipid extraction and radioactivity detection. *In vivo* RCT efficiency was calculated as the percentage of radioactivity in the plasma, liver, or feces to total radioactivity injected.

### 2.6. Measurement of Serum Lipid Levels

Blood samples from the retro-orbital plexus of experimental mice were collected into EDTA-coated tubes. Plasma levels of triglycerides (TG), total cholesterol (TC), HDL cholesterol (HDL-C), and low-density lipoprotein cholesterol (LDL-C) were quantified by enzymatic methods utilizing commercial kits (Nanjing Jiancheng Biotech Inc., Jiangsu, China).

### 2.7. Evaluation of Hepatic Lipid Levels

The hepatic tissues were isolated from euthanized mice, washed in PBS, dried on filter papers, weighed, fixed with 4% formaldehyde at room temperature for 24 h, and then homogenized. The TC and TG levels were measured using commercial kits (Jiancheng Biotech) according to the manufacturer's protocol.

### 2.8. Cell Culture and Treatment

THP-1 cells were purchased from American Type Culture Collection (ATCC, USA) and maintained in RPMI 1640 medium supplemented with 1% penicillin-streptomycin (Beyotime, Shanghai, China) and 10% FBS. Cells were cultured at 37°C in a humidified atmosphere of 5% CO_2_. To stimulate the differentiation of monocytes into macrophages, THP-1 cells were treated with 160 nM phorbol 12-myristate 13-acetate (PMA, Sigma-Aldrich, St. Louis, MO, USA) for 24 h. THP-1 macrophages were then incubated with 50 *μ*g/mL ox-LDL (Yiyuan biotechnology) for 48 h to transform into foam cells. THP-1 macrophage-derived foam cells were seeded in 24-well plate and grown to 80% confluence. Subsequently, these cells were treated with BCA dissolved in 1% DMSO or vehicle. To interfere with the PPAR*γ*/LXR*α* or PPAR*γ*/HO-1 pathway, THP-1 macrophage-derived foam cells were transfected with 50 nM siRNAs against PPAR*γ*, LXR*α*, or HO-1 (GenePharma, Shanghai, China) for 24 h. After transfection, the cells were treated with BCA for another 24 h. A scrambled siRNA was used as a nontargeting negative control. Western blot was performed to determine transfection efficiency.

### 2.9. MTT Assay

The cytotoxicity of BCA on THP-1 macrophage-derived foam cells was evaluated using MTT assay. Briefly, cells were seeded into 96-well plates at a density of 5 × 10^4^cells/well. When cells were 80% confluent, the cells were treated with different doses of BCA (5, 10, 20, or 40 *μ*M) for 24 h or 20 *μ*M BCA for various times (6, 12, 24, or 48 h). Then, cells were incubated with 20 *μ*L of 4 mg/mL MTT solution (Sigma-Aldrich) at 37°C for 4 h. The medium was carefully removed and each well was added with 150 *μ*L DMSO, followed by gentle shake. The optical density (OD) value of each well was measured at 490 nm.

### 2.10. Cholesterol Efflux Assay

Cholesterol efflux assay was conducted as described previously [24]. Briefly, THP-1 macrophages and MPMs isolated from apoE^−/−^ mice were treated with 50 *μ*g/mL of ox-LDL and 5 *μ*Ci/mL [^3^H]-cholesterol for 48 h. Then, cells were rinsed with PBS and incubated with serum-free medium containing 0.1% BSA for 2 h for equilibration. Then, cells were washed in PBS again and maintained in RPMI 1640 medium containing 0.5% BSA and 25 *μ*g/mL apoA-I (Sigma-Aldrich) or 50 *μ*g/mL HDL (Sigma-Aldrich) at 37°C overnight. The medium and cells were collected for radioactivity detection using a liquid scintillation counter. The efflux capacity was quantified as the ratio of [^3^H] activity in the medium to total [^3^H] activity (medium + cells).

### 2.11. Evaluation of Intracellular Lipid Droplets by Oil Red O Staining

THP-1 macrophage-derived foam cells were washed with PBS and fixed in 4% paraformaldehyde for 5 min. After several rinses, they were stained with prepared Oil Red O working solution (Yiyuan biotechnology) in the dark at 37 °C for 20 min, and further destained with 60% isopropanol for 10 sec. Finally, the Oil Red O-positive cells (red) were photographed using a fluorescent microscope (Olympus BX50) at ×400 magnification.

### 2.12. Detection of Intracellular Cholesterol Contents by High-Performance Liquid Chromatography (HPLC)

HPLC assay was performed as previously described by us [24]. MPMs and THP-1 macrophage-derived foam cells were broken in 1 mL 0.9% NaCl using an ultrasonic processor (Scientz, Zhejiang, China) under ice bath. Following centrifugation (12,000 rpm, 5 min), the supernatants were collected and vortexed. Cholesterol was extracted using isopropanol/hexane (2:3, *V*/*V*) and dissolved in isopropanol (50 mg/mL). Cholesterol standard calibration solution ranging from 0 to 50 mg/mL was prepared. The reaction mixture (500 mM MgCl_2_, 500 mM TriS-HCl (PH = 7.4), 10 mM dithiothreitol, and 5% NaCl) was supplemented into 100 *μ*L of the sample or cholesterol standard calibration solution. 0.4 U cholesterol oxidase combined with 0.4 U cholesterol esterase was supplemented to detect TC content. The FC content was measured without adding cholesterol esterase. After incubating at 37°C for 30 min, the reaction was terminated. The supernatant was then collected and detected by a high-performance liquid chromatographer (LC10AVP, Shimadzu, Japan). The column was eluted using isopropanol : heptane : acetonitrile (35 : 12 : 53) at a flow rate of 1 mL/min for 8 min. Absorbance at 226 nm was monitored. Data analyses were conducted using TotalChrom software (PerkinElmer).

### 2.13. Dil-ox-LDL Uptake Assay

THP-1 macrophage-derived foam cells were treated with BCA or PBS, followed by incubation with 10 *μ*g/mL Dil-ox-LDL (Yiyuan biotechnology) at 37°C for 4 h. After washing with PBS three times, the cells were viewed and imaged by a fluorescence inverted microscope (Olympus BX50) at ×200 magnification.

### 2.14. RNA Isolation and Quantitative Real-Time PCR (qRT-PCR)

The TRIzol reagent kit (Invitrogen, CA, USA) was used to extract total RNA from the obtained tissues and cultured cells. The extracted RNA was then purified and quantified using the Nanodrop 3000 (ThermoFisher, Scotts Valley, CA, USA). The first-strand complementary DNA was synthesized utilizing a high-capacity cDNA reverse transcription kit (Takara, Kyoto, Japan). qRT-PCR was conducted using SYBR® Premix Ex TaqTM II reagent kit (Takara) on an ABI 7900HT Fast Real-Time PCR System (Applied Biosystems, Foster City, CA, USA) for 40 cycles (95°C for 2 min, 95°C for 10 sec, and 60°C for 30 sec). Melting curve analysis was used to evaluate the specificity of all PCR products. Relative gene expression was quantitated using the 2^−ΔΔ^Ct method. The sequences of mRNA primers were designed and synthesized by Shanghai Sangon Biotech Co., Ltd (Shanghai, China). The primers were listed in Supplementary Table 1. *β*-Actin was used as an internal control.

### 2.15. Western Blot Analysis

The cells and tissues were lysed with the RIPA buffer (Beyotime) containing 0.1 mmol/L PMSF (Beyotime) on ice for 15 min, and total proteins were isolated by centrifugation at 12,000 rpm for 10 min at 4°C. The concentration of protein extracts was measured by a BCA Assay Kit (Beyotime). Then, the protein extracts were subjected to SDS-PAGE and transferred to a polyvinylidene difluoride (PVDF) membrane (Merck Millipore, MA, USA). After blocking with 5% skim milk at 4°C for 4 h, the membranes were immunoblotted with mouse monoclonal antibody against ABCA1 (ab18180, 1:500, Abcam, Cambridge, MA, USA), rabbit monoclonal antibody against ABCG1 (ab52617, 1:1000, Abcam), rabbit monoclonal antibody against CD36 (ab133625, 1:500, Abcam), mouse polyclonal antibody against SR-A (AF1797, 1:1000, R&D Systems, MN, USA), rabbit monoclonal antibody against LXR*α* (ab176323, 1:500, Abcam), rabbit monoclonal antibody against PPAR*γ* (ab178860, 1:500, Abcam), and rabbit monoclonal antibody against HO-1 (#70081, 1:1000, CST, Danvers, MA, USA) with gentle shaking overnight at 4°C. After a series of rinses with PBST, the membranes were further incubated with HRP-conjugated secondary antibodies (1:5000, CWbio, Beijing, China). The protein bands were visualized with BeyoECL Plus kit (Beyotime) and analyzed using Gel-Pro software 4.0. *β*-Actin was used as an internal control.

### 2.16. Enzyme-Linked Immunosorbent Assay (ELISA)

Serum samples and cell culture supernatant were collected. Then, the commercial ELISA kits (R&D Systems) were used to quantify the levels of tumor necrosis factor-*α* (TNF-*α*), interleukin (IL)-1*β*, and IL-6 according to the manufacturer's protocol. The absorbance at 450 nm was detected using the iMark™ Microplate Reader (Bio-Rad, Hercules, CA, USA).

### 2.17. Statistical Analysis

All data are expressed as the mean ± standarddeviation (S.D.) from at least three independent experiments. All statistical analyses among groups were performed by either one-way ANOVA followed by Tukey's multiple comparison test or unpaired Student's *t*-test, using GraphPad Prism 8.0 software (CA, USA). A value of *P* < 0.05 was considered as statistical significance.

## 3. Results

### 3.1. BCA Inhibits Atherosclerotic Plaque Formation in apoE^−/−^ Mice

To determine the effects of BCA on atherogenesis, apoE^−/−^ mice fed a Western diet were administered with BCA or vehicle. A similar weight gain was observed in two groups over the course of the study (Figure 1(b)). There were no significant differences in the serum levels of ALT, AST, Bun, and Scr between two groups, indicating that BCA at currently used dose has no hepatotoxicity and nephrotoxicity (Figure 1(c)). Notably, treatment with BCA obviously decreased the size of atherosclerotic lesions in the aortic arch (Figure 1(d)) and *en face* aorta (Figure 1(e)). BCA also reduced lesion area and lipid deposition in the cross-sections of the aortic root, as evidenced by the HE and Oil Red O staining (Figure 1(f)). However, Masson staining showed that BCA did not alter collagen content within the plaques, indicating that it has no effect on plaque stability (Figure 1(f)). Altogether, these *in vivo* observations suggest that BCA can inhibit the development of atherosclerosis.

### 3.2. BCA Promotes RCT and Improve Plasma Lipid Profile in apoE^−/−^ Mice

**Figure 2:**
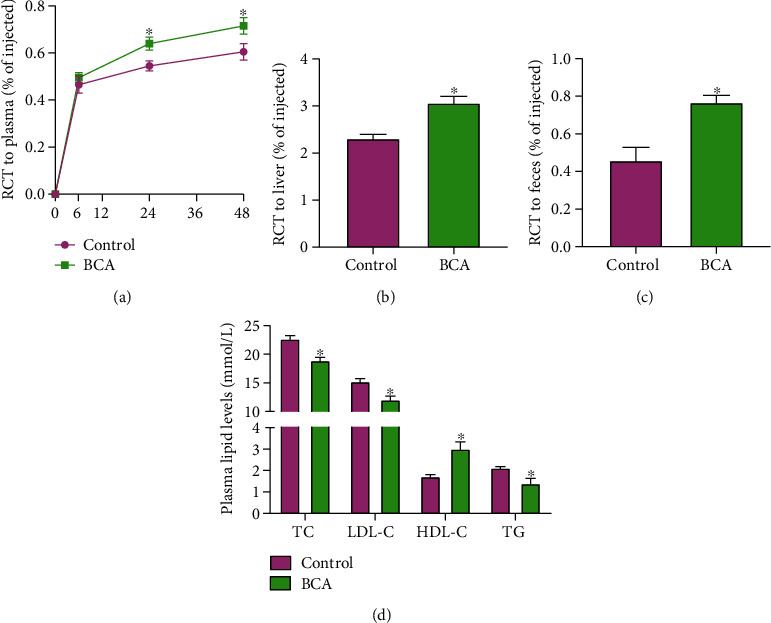
BCA improves RCT and plasma lipid profile in apoE^−/−^ mice. (a–c) J774 macrophages loaded with [^3^H]-cholesterol and acetylated LDL were injected into the abdominal cavity of apoE^−/−^ mice (*n* = 5 per group). The radioactivity in the plasma, liver, and feces were measured by a liquid scintillation counter. (d) Plasma levels of TC, LDL-C, HDL-C, and TG were measured using enzymatic methods (*n* = 10 per group). Data are expressed as mean ± SD. ^∗^*P* < 0.05 vs. control group.

Given that RCT is a major pathway to eliminate excessive cholesterol from the body and exerts an atheroprotective effect [25], we next tested the impact of BCA on RCT. As expected, administration of BCA markedly increased [^3^H]-cholesterol content in the plasma, liver, and feces (Figures 2(a)–2(c)), revealing a beneficial effect of BCA on RCT. Accordingly, BCA treatment led to a remarkable increase in plasma HDL-C levels and a decrease in TC, LDL-C, and TG levels (Figure 2(d)), suggesting that BCA can ameliorate hyperlipidemia. Notably, hepatic TC and TG levels trended higher in BCA group compared with control group but were not significantly different, indicating that administration of BCA has no impact on fat content in the liver and does not lead to fatty liver (Supplementary Fig. 1).

### 3.3. BCA Accelerates Macrophage Cholesterol Efflux and Alleviates Intracellular Lipid Accumulation

**Figure 3:**
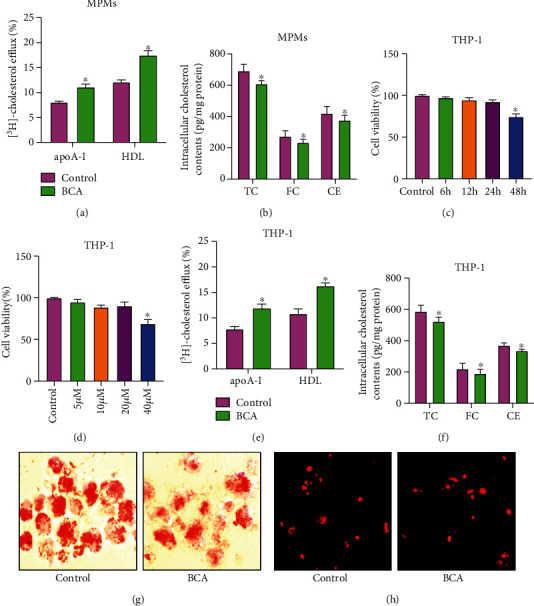
Effects of BCA on cholesterol efflux and lipid accumulation in macrophages. (a) MPMs were incubated with [^3^H]-cholesterol for 48 h, and cholesterol efflux to apoA-I or HDL was detected using a liquid scintillation counter. (b) Detection of intracellular TC, FC, and CE contents by HPLC. (c, d) THP-1 macrophage-derived foam cells were treated with 20 *μ*M BCA for different time periods or various doses of BCA for 24 h. MTT assay was used to evaluate cell viability. (e) THP-1 macrophage-derived foam cells were labeled with [^3^H]-cholesterol and then treated with 20 *μ*M BCA for 24 h. The efflux of cholesterol to apoA-I or HDL was then quantified. (f) Analysis of the levels of intracellular TC, FC, and CE using HPLC assay. (g) Representative images of Oil red O staining (400x). (h) Representative images of Dil-ox-LDL uptake (200x). Data are expressed as mean ± SD from at least three independent experiments. ^∗^*P* < 0.05 vs. control group.

Cholesterol efflux, the first and rate-limiting step of RCT, is essential for the formation and maturation of HDL particles [26]. We speculated that BCA-mediated improvement of plasma lipid profile is attributed to its ability to stimulate cholesterol efflux. To this end, we isolated MPMs from apoE^−/−^ mice and found that BCA treatment significantly increased cholesterol efflux from MPMs to apoA-I and HDL (Figure 3(a)) and decreased intracellular cholesterol contents (Figure 3(b)). To further confirm this effect, THP-1 macrophage-derived foam cells were exposed to BCA as indicated time and concentrations. The MTT results showed that treatment with 20 *μ*M BCA for 24 h had no effects on cell viability (Figures 3(c) and 3(d)). Thus, this concentration and time was selected for the subsequent *in vitro* experiments. Consistent with the findings obtained from MPMs, THP-1 macrophage-derived foam cells treated with BCA displayed a significant increase in cholesterol efflux to apoA-I and HDL (Figure 3(e)). Accordingly, BCA treatment caused a dramatic decrease in intracellular TC, FC, and CE amounts (Figure 3(f)), as well as a reduction of lipid droplets in THP-1 macrophage-derived foam cells (Figure 3(g)), indicating an inhibitory effect of BCA on lipid accumulation. In addition to increased cholesterol efflux, decreased cholesterol uptake contributes to prevention of lipid accumulation [26]. Unexpectedly, there was no difference in the uptake of Dil-ox-LDL by THP-1 macrophage-derived foam cells between the control group and BCA group (Figure 3(h)). Together, these results suggest that BCA inhibits lipid accumulation by promoting cholesterol efflux from macrophages.

### 3.4. BCA Upregulates ABCA1 and ABCG1 Expression In Vivo and In Vitro

**Figure 4:**
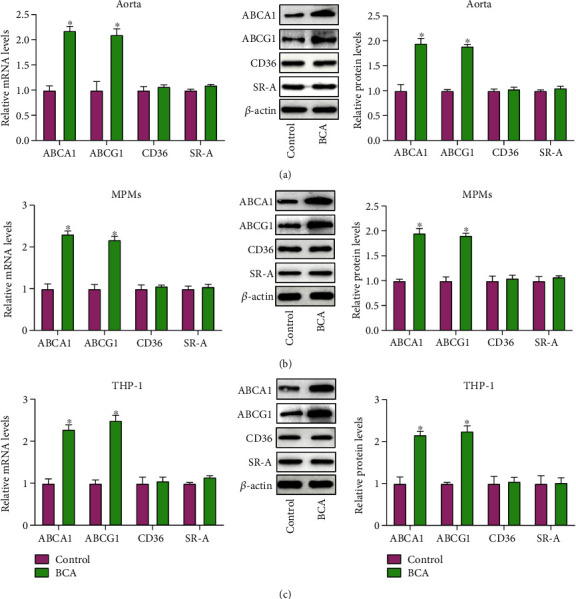
Effects of BCA on ABCA1, ABCG1, CD36, and SR-A expression. (a) The qRT-PCR and Western blot analyses of ABCA1, ABCG1, CD36, and SR-A expression in the aorta from apoE^−/−^ mice (*n* = 10). (b) Measurement of ABCA1, ABCG1, CD36, and SR-A expression in MPMs by qRT-PCR and Western blot (*n* = 5). (c) THP-1 macrophage-derived foam cells were incubated with 20 *μ*M BCA for 24 h, followed by qRT-PCR and Western blot assays (*n* = 3). Data are expressed as mean ± SD. ^∗^*P* < 0.05 vs. control group.

It is well known that ABCA1 and ABCG1 mediate the efflux of cholesterol, while CD36 and SR-A are responsible for cholesterol uptake [27, 28]. To gain insights into potential mechanisms, qRT-PCR and Western blot were used to measure the expression of these agents. Our results demonstrated that administration of BCA significantly increased the mRNA and protein levels of ABCA1 and ABCG1 in mouse aorta (Figure 4(a)) and MPMs (Figure 4(b)). A similar result was observed in THP-1 macrophage-derived foam cells treated with BCA (Figure 4(c)). However, the expression of CD36 and SR-A was unchangeable in response to BCA both *in vivo* (Figures 4(a) and 4(b)) and *in vitro* (Figure 4(c)). Taken together, these observations suggest that BCA upregulates ABCA1 and ABCG1 expression to promote macrophage cholesterol efflux and inhibit lipid accumulation.

### 3.5. The PPAR*γ*/LXR*α* and PPAR*γ*/HO-1 Pathways are Involved in BCA-Induced Upregulation of ABCA1 and ABCG1 Expression

**Figure 5:**
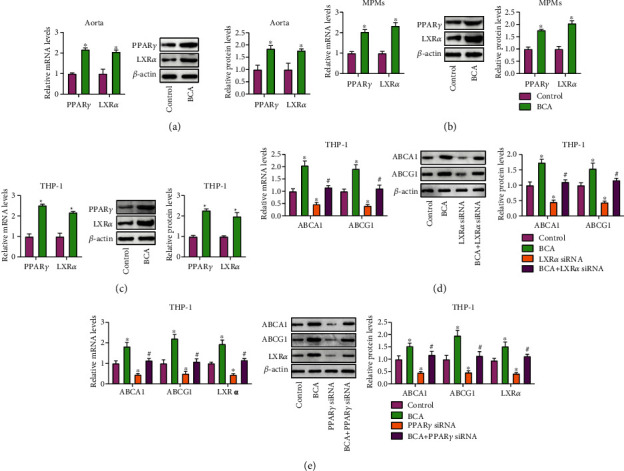
Involvement of the PPAR*γ*/LXR*α* pathway in BCA-induced upregulation of ABCA1 and ABCG1 expression. (a) Evaluation of PPAR*γ* and LXR*α* expression in the aorta from apoE^−/−^ mice (*n* = 10). (b) Detection of PPAR*γ* and LXR*α* expression in MPMs from apoE^−/−^ mice (*n* = 5). (c) THP-1 macrophage-derived foam cells were treated with 20 *μ*M BCA or vehicle for 24 h. The expression of PPAR*γ* and LXR*α* was determined by qRT-PCR and Western blot (*n* = 3). (d, e) THP-1 macrophage-derived foam cells were transfected with 50 nM of LXR*α* siRNA or PPAR*γ* siRNA for 24 h, followed by incubation with or without 20 *μ*M BCA for another 24 h. Both qRT-PCR and Western blot were performed to detect LXR*α*, ABCA1, and ABCG1 expression (*n* = 3). Data are expressed as mean ± SD. ^∗^*P* < 0.05 vs. control group; ^**#**^*P* < 0.05 vs. BCA group.

The PPAR*γ*/LXR*α* pathway is known to play a central role in stimulating ABCA1 and ABCG1 transcription [29, 30]. Next, we explored whether this signaling axis is involved in BCA-induced upregulation of these two transporters' expression. The qRT-PCR and Western blot results showed that treatment with BCA dramatically increased PPAR*γ* and LXR*α* expression in the aorta (Figure 5(a)), MPMs (Figure 5(b)), and THP-1 macrophage-derived foam cells (Figure 5(c)). Subsequently, THP-1 macrophage-derived foam cells were transfected with siRNAs against LXR*α* or PPAR*γ*, followed by treatment with or without BCA. As illustrated in Supplementary Fig. 2, the endogenous expression of LXR*α* and PPAR*γ* was effectively silenced by LXR*α* siRNA and PPAR*γ* siRNA, respectively. Importantly, BCA-induced upregulation of ABCA1 and ABCG1 expression was partially reversed by siRNAs against LXR*α* and PPAR*γ* (Figures 5(d) and 5(e)). Knockdown of PPAR*γ* with siRNA also abrogated the effect of BCA on LXR*α* expression (Figure 5(e)). All these findings support the notion that BCA increases ABCA1 and ABCG1 expression, at least in part, by activating the PPAR*γ*/LXR*α* pathway.

**Figure 6:**
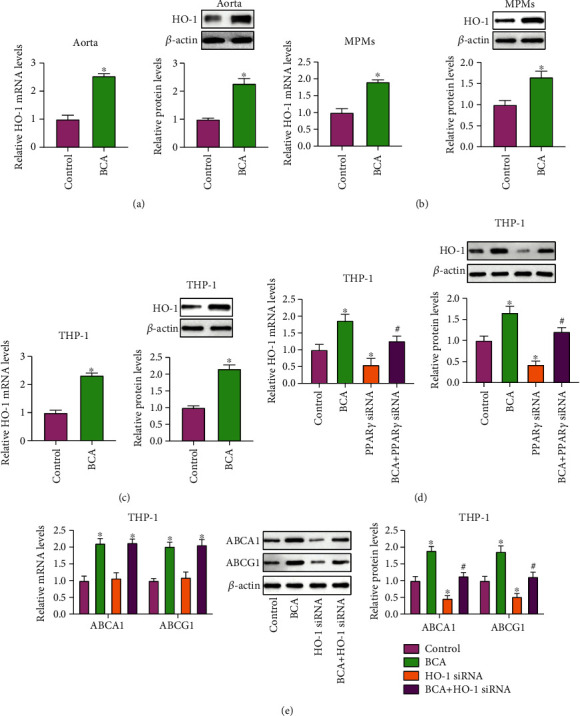
BCA-induced enhancement of ABCA1 and ABCG1 expression is mediated by the PPAR*γ*/HO-1 pathway. (a) The mRNA and protein levels of HO-1 in the aorta from apoE^−/−^ mice were detected by qRT-PCR and Western blot, respectively (*n* = 10). (b) Analysis of HO-1 expression in MPMs using qRT-PCR and Western blot (*n* = 5). (c) THP-1 macrophage-derived foam cells were treated with 20 *μ*M BCA or vehicle for 24 h. HO-1 expression was then measured by qRT-PCR and Western blot. (d, e) THP-1 macrophage-derived foam cells were transfected with 50 nM of PPAR*γ* siRNA or HO-1 siRNA for 24 h, which was followed by treatment with or without 20 *μ*M BCA for the same time. Both qRT-PCR and Western blot were performed to detect the expression of HO-1, ABCA1, and ABCG1 (*n* = 3). Data are expressed as mean ± SD. ^∗^*P* < 0.05 vs. control group; ^**#**^*P* < 0.05 vs. BCA group.

In addition to LXR*α*, HO-1 is a downstream effector molecule of PPAR*γ*. It has been reported that HO-1 increases the protein stability of ABCA1 and ABCG1 by inhibiting calpain-mediated proteolysis [31, 32]. It is likely that BCA-induced protective effects on the expression of ABCA1 and ABCG1 is also mediated by the PPAR*γ*/HO-1 pathway. To test this possibility, we first detected HO-1 expression using qRT-PCR and Western blot and found that BCA treatment dramatically increased the mRNA and protein levels of HO-1 in mouse aorta (Figure 6(a)), MPMs (Figure 6(b)), and THP-1 macrophage-derived foam cells (Figure 6(c)). THP-1 macrophage-derived foam cells were then transfected with siRNAs against PPAR*γ* or HO-1, which was followed by incubation with or without BCA. PPAR*γ* knockdown abolished the effect of BCA on HO-1 expression (Figure 6(d)). The Western blot results confirmed efficient knockdown of HO-1 expression by HO-1 siRNA (Supplementary Fig. 2). Transfection with HO-1 siRNA successfully inhibited BCA-induced increase in protein expression of ABCA1 and ABCG1 without affecting their mRNA levels (Figure 6(e)), suggesting that activation of the PPAR*γ*/HO-1 pathway is also involved in induction of ABCA1 and ABCG1 expression by BCA.

### 3.6. BCA Inhibits Inflammatory Response In Vivo and In Vitro

**Figure 7:**
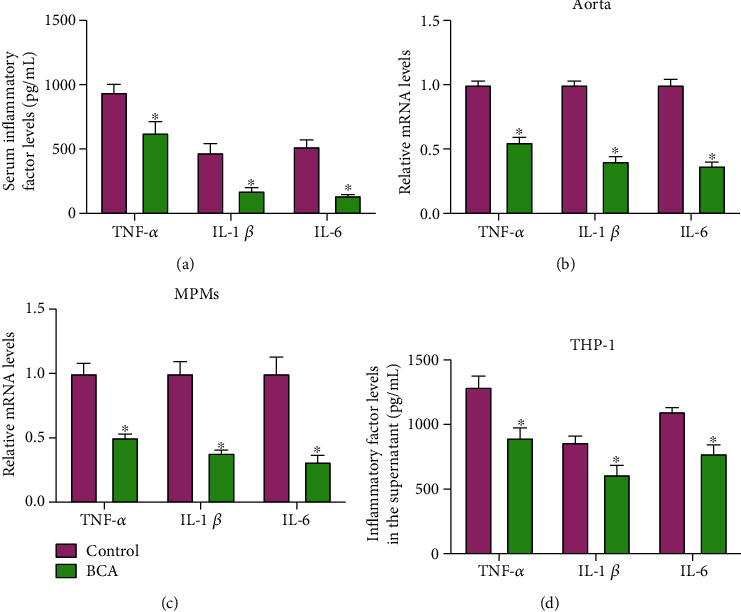
BCA inhibits inflammatory response *in vivo* and *in vitro*. (a) Serum levels of TNF-*α*, IL-1*β*, and IL-6 were detected using ELISA assay in apoE^−/−^ mice (*n* = 10). (b) Expression of TNF-*α*, IL-1*β*, and IL-6 mRNA in the aorta of apoE^−/−^ mice was measured by qRT-PCR (*n* = 10). (c) The mRNA levels of TNF-*α*, IL-1*β*, and IL-6 were detected using qRT-PCR in MPMs from apoE^−/−^ mice (*n* = 5). (d) THP-1 macrophage-derived foam cells were treated with 20 *μ*M BCA or vehicle for 24 h. The cell culture supernatant was collected to examine the levels of TNF-*α*, IL-1*β*, and IL-6 by ELISA (*n* = 3). Data are expressed as mean ± SD. ^∗^*P* < 0.05 vs. control group.

In addition to lipid metabolism disorder, inflammatory response is closely associated with the occurrence and development of atherosclerosis [33]. As illustrated in Figure 7(a), administration of BCA diminished serum TNF-*α*, IL-1*β*, and IL-6 levels. Accordingly, the mRNA levels of TNF-*α*, IL-1*β*, and IL-6 were significantly decreased in the aorta (Figure 7(b)) and MPMs (Figure 7(c)) isolated from BCA-treated apoE^−/−^ mice. In addition, the secretion of these proinflammatory cytokines was inhibited by BCA in THP-1 macrophage-derived foam cells (Figure 7(d)). These results suggest that blockade of inflammatory response is another important mechanism underlying the atheroprotective action of BCA.

### 3.7. The PPAR*γ*/LXR*α* and PPAR*γ*/HO-1 Pathways are Required for the Inhibitory Effect of BCA on Inflammatory Response

**Figure 8:**
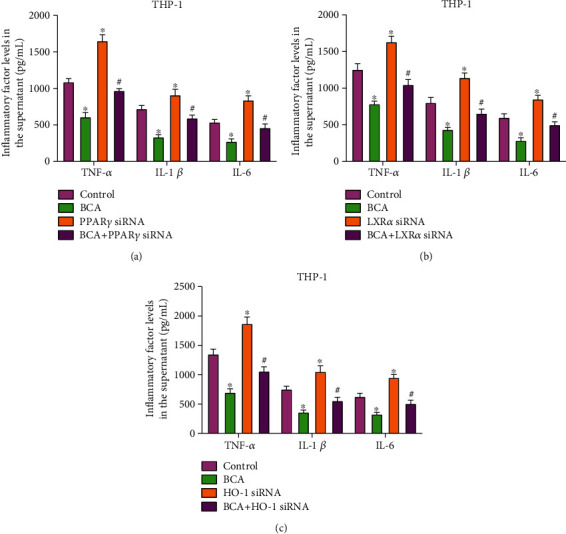
Involvement of the PPAR*γ*/LXR-*α* and PPAR*γ*/HO-1 pathways in the inhibitory effect of BCA on inflammatory response. (a–c) THP-1 macrophage-derived foam cells were transfected with 50 nM siRNAs against PPAR*γ*, LXR*α*, or HO-1 for 24 h, followed by incubation with or without 20 *μ*M BCA for another 24 h. ELISA was used to measure the levels of TNF-*α*, IL-1*β*, and IL-6 in the cell culture supernatant. Data are expressed as mean ± SD from three independent experiments. ^∗^*P* < 0.05 vs. control group; ^**#**^*P* < 0.05 vs. BCA group.

Activation of the PPAR*γ*/LXR*α* and PPAR*γ*/HO-1 pathways also contributes to prevention of inflammatory response in multiple cell types [16, 34]. Given BCA as a positive regulator of PPAR*γ* expression [35, 36], we inferred that the anti-inflammatory effect of BCA is mediated by these two signaling pathways. To do so, THP-1 macrophage-derived foam cells were pretreated with siRNAs against PPAR*γ*, LXR*α*, or HO-1, followed by incubation with or without BCA. As expected, treatment with BCA alone led to a significant decrease in TNF-*α*, IL-1*β*, and IL-6 secretion, and this decrease was prevented by PPAR*γ* siRNA, LXR*α* siRNA, and HO-1 siRNA (Figures 8(a)–8(c)). These results suggest that BCA activates the PPAR*γ*/LXR*α* and PPAR*γ*/HO-1 pathways to antagonize inflammation.

## 4. Discussion

Although statins and other new therapeutic agents for atherosclerosis have been successfully applied, it is needed to find new drugs to meet the unmet clinic demand for its high morbidity and mortality [37, 38]. There is increasing evidence that plant flavonoid isoflavones exert a beneficial effect in lipid metabolism, inflammation, and atherosclerosis [39, 40]. BCA, a bioactive isoflavone extracted from many herbal products, plays a protective role in CVD [41, 42]. However, whether BCA affects atherogenesis remains to be determined. In the present study, we found that BCA decreased atherosclerotic lesion size and alleviated lipid deposition within plaques in apoE^−/−^ mice, suggesting a favorable role in the development of atherosclerosis.

Atherosclerosis is regarded as a lipid-driven inflammatory disease [43, 44]. It has been reported that administration of BCA increases plasma HDL-C levels and decreases LDL-C and TC levels in animal models of hyperlipidemia [45]. BCA also reduces proinflammatory cytokine secretion in human umbilical vein endothelial cells and primary rat chondrocytes [46, 47]. Similarly, our data showed that BCA improved plasma lipid profile, promoted overall RCT efficiency, and decreased serum TNF-*α*, IL-1*β*, and IL-6 levels in apoE^−/−^ mice. Thus, BCA protects against atherosclerosis by improving lipid metabolism and inhibiting inflammatory response.

Lipid accumulation leads to foam cell formation within the arterial wall, an early event in the development of atherosclerosis [48]. Here, we found that BCA-treated macrophages exhibited a significant decrease in lipid droplets and intracellular cholesterol amounts, indicating this isoflavone as a potent suppressor of lipid accumulation. Intracellular cholesterol level depends on the dynamic balance between cholesterol internalization and cholesterol efflux. It is now well accepted that CD36 and SR-A are responsible for cholesterol uptake by macrophages, while ABCA1 and ABCG1 mediate intracellular cholesterol efflux [49]. Mutations in ABCA1 gene cause Tangier disease, which is characterized by extremely low plasma HDL-C levels and premature atherosclerosis [50]. Double knockout of ABCA1 and ABCG1 genes in apoE^−/−^ mice leads to more atherosclerotic lesions than single knockout mice [51]. In this study, we observed that BCA had no effect on Dil-ox-LDL uptake but promoted cholesterol efflux from MPMs and THP-1 macrophage-derived foam cells, in agreement with a previous report showing that BCA has a beneficial effect on cholesterol efflux from RAW 264.7 macrophages [45]. Accordingly, treatment with BCA upregulated ABCA1 and ABCG1 expression without altering SR-A and CD36 levels. These findings suggest that promoting ABCA1- and ABCG1-dependent cholesterol efflux is an important mechanism for BCA-induced alleviation of lipid accumulation.

LXR*α* is the most important transcriptional factor to induce ABCA1 and ABCG1 expression. PPAR*γ* acts as an upstream molecule of LXR*α*. Activation of the PPAR*γ*/LXR*α* signaling pathway has been shown to increase ABCA1 and ABCG1 expression and thereby mitigate atherosclerosis [52]. HO-1, a key enzyme involving heme catabolism, is abundantly expressed in macrophages. Deletion of HO-1 promotes the development of atherosclerosis in apoE^−/−^ mice [53], whereas its overexpression leads to decreased atherosclerotic lesions [54]. Unlike LXR*α*, HO-1 can stabilize ABCA1 and ABCG1 proteins by reducing calpain activity. It has been reported that administration of kaempferol and Tanshinone IIA markedly increases the protein levels of ABCA1 and ABCG1 in a HO-1-dependent manner in THP-1 macrophages [15, 32]. Our results showed that treatment with BCA increased PPAR*γ*, LXR*α*, and HO-1 expression in the aorta and macrophages. Importantly, BCA-induced upregulation of ABCA1 and ABCG1 expression was reversed by pretreatment with siRNAs against PPAR*γ*, LXR*α*, and HO-1 in THP-1 macrophage-derived foam cells. Thus, BCA increases ABCA1 and ABCG1 expression through two molecular mechanisms. On one hand, BCA activates the PPAR*γ*/LXR*α* pathway to stimulate transcription of the two ABC transporter genes. On the other hand, BCA inhibits protein degradation through the PPAR*γ*/HO-1 pathway.

In addition to lipid metabolism, LXR*α* and HO-1 are associated with inflammatory response. A recent study showed that LXR*α* is expressed at higher levels in healthy people compared with atherosclerosis patients, and its overexpression polarizes macrophages towards an anti-inflammatory M2 phenotype [55]. Treatment with saikosaponin a (SSa) inhibits lipopolysaccharide-induced proinflammatory cytokine production in primary mouse macrophages by upregulating LXR*α* expression [56]. HO-1 deletion in myeloid cells promotes the polarization of proinflammatory M1 macrophages both *in vivo* and *ex vivo*, while transgenic overexpression of HO-1 favors an M2 anti-inflammatory phenotype [57], revealing a regulatory role of HO-1 in macrophage inflammation and atherogenesis. Additionally, activation of PPAR*γ* has been shown to reduce proinflammatory cytokine secretion by upregulating HO-1 expression in multiple cell types [58, 59]. The present study demonstrated that knockdown of PPAR*γ*, LXR*α*, and HO-1 with siRNAs abolished the inhibitory effect of BCA on TNF-*α*, IL-1*β*, and IL-6 secretion from THP-1 macrophage-derived foam cells. Thus, BCA suppresses inflammatory response by activating the PPAR*γ*/LXR*α* and PPAR*γ*/HO-1 pathways. However, it remains to be determined whether BCA directly or indirectly regulates PPAR*γ* expression.

In summary, the present study has revealed a beneficial role of BCA in atherosclerosis. Mechanistically, BCA promotes ABCA1- and ABCG1-dependent cholesterol efflux and inhibits inflammatory response by activating the PPAR*γ*/LXR*α* and PPAR*γ*/HO-1 pathways. Thus, BCA could be developed as a promising antiatherogenic drug in the future.

## Data Availability

The data used to support the findings of this study are available from the corresponding author upon request.

## Supplementary Materials

Supplementary Materials Supplementary Figure 1: BCA has no effect on fat contents in the liver. Hepatic tissues were isolated from apoE^−/−^ mice, and the levels of TC and TG were detected using commercial kits (*n* = 10). Data are expressed as mean ± SD. Supplementary Figure 2: evaluation of siRNA transfection efficiency. (a–c) THP-1 macrophage-derived foam cells were transfected with 50 nM of scrambled siRNA, LXR*α* siRNA, PPAR*γ* siRNA, or HO-1 siRNA for 24 h, followed by Western blot assay for LXR*α*, PPAR*γ*, and HO-1 expression. Data are expressed as mean ± SD from three independent experiments. ^∗^*P* < 0.05 vs. control group. Supplementary Table 1: the primer sequences used in qRT-PCR.

## References

[1] Mahjoubin-Tehran M., Kovanen P., Xu S., Jamialahmadi T., Sahebkar A. (2020). Cyclodextrins: potential therapeutics against atherosclerosis. *Pharmacology & Therapeutics*.

[2] Schaftenaar F., Frodermann V., Kuiper J., Lutgens E. (2016). Atherosclerosis. *Current Opinion in Lipidology*.

[3] Zhu Y., Xian X., Wang Z. (2018). Research progress on the relationship between atherosclerosis and inflammation. *Biomolecules*.

[4] Choi S. H., Sviridov D., Miller Y. I. (2017). Oxidized cholesteryl esters and inflammation. *Biochimica et biophysica acta Molecular and cell biology of lipids*.

[5] Kuhnast S., van der Tuin S. J. L., van der Hoorn J. W. A. (2015). Anacetrapib reduces progression of atherosclerosis, mainly by reducing non-HDL-cholesterol, improves lesion stability and adds to the beneficial effects of atorvastatin. *European Heart Journal*.

[6] Sabatine M. S., Giugliano R. P., Keech A. C. (2017). Evolocumab and clinical outcomes in patients with cardiovascular disease. *The New England journal of medicine*.

[7] Rohatgi A., Khera A., Berry J. D. (2014). HDL cholesterol efflux capacity and incident cardiovascular events. *The New England journal of medicine*.

[8] Westerterp M., Fotakis P., Ouimet M. (2018). Cholesterol efflux pathways suppress inflammasome activation, NETosis, and atherogenesis. *Circulation*.

[9] Wang G., Gao J. H., He L. H. (2020). Fargesin alleviates atherosclerosis by promoting reverse cholesterol transport and reducing inflammatory response. *Biochimica et Biophysica Acta (BBA) - Molecular and Cell Biology of Lipids*.

[10] Peng Y., Xu J., Zeng Y., Chen L., Le Xu X. (2019). Polydatin attenuates atherosclerosis in apolipoprotein E-deficient mice: role of reverse cholesterol transport. *Phytomedicine*.

[11] Guo S., Tian H., Dong R. (2016). Exogenous supplement of N-acetylneuraminic acid ameliorates atherosclerosis in apolipoprotein E-deficient mice. *Atherosclerosis*.

[12] Chawla A., Boisvert W. A., Lee C. H. (2001). A PPAR gamma-LXR-ABCA1 pathway in macrophages is involved in cholesterol efflux and atherogenesis. *Molecular cell*.

[13] Fessler M. B. (2018). The challenges and promise of targeting the Liver X Receptors for treatment of inflammatory disease. *Pharmacology & therapeutics*.

[14] Vijayan V., Wagener F. A. D. T. G., Immenschuh S. (2018). The macrophage heme-heme oxygenase-1 system and its role in inflammation. *Biochemical pharmacology*.

[15] Liu Z., Wang J., Huang E. (2014). Tanshinone IIA suppresses cholesterol accumulation in human macrophages: role of heme oxygenase-1. *Journal of lipid research*.

[16] Xu J., Zhu Y. T., Wang G. Z. (2015). The PPAR*γ* agonist, rosiglitazone, attenuates airway inflammation and remodeling via heme oxygenase-1 in murine model of asthma. *Acta pharmacologica Sinica*.

[17] Abdalla H. B., Napimoga M. H., Lopes A. H. (2020). Activation of PPAR-*γ* induces macrophage polarization and reduces neutrophil migration mediated by heme oxygenase 1. *International immunopharmacology*.

[18] Sarfraz A., Javeed M., Shah M. A. (2020). Biochanin A: a novel bioactive multifunctional compound from nature. *The Science of the total environment*.

[19] Zhao X., Tang X., Guo N. (2018). Biochanin a enhances the defense against Salmonella enterica infection through AMPK/ULK1/mTOR-mediated autophagy and extracellular traps and reversing SPI-1-dependent macrophage (M*Φ*) M2 polarization. *Frontiers in cellular and infection microbiology*.

[20] Yu C., Zhang P., Lou L., Wang Y. (2019). Perspectives regarding the role of biochanin A in humans. *Frontiers in pharmacology*.

[21] Wang W., Tang L., Li Y., Wang Y. (2015). Biochanin A protects against focal cerebral ischemia/reperfusion in rats via inhibition of p38-mediated inflammatory responses. *Journal of the neurological sciences*.

[22] Park H. S., Hur H. J., Kim S. H. (2016). Biochanin A improves hepatic steatosis and insulin resistance by regulating the hepatic lipid and glucose metabolic pathways in diet-induced obese mice. *Molecular nutrition & food research*.

[23] Ren K., Li H., Zhou H. F. (2019). Mangiferin promotes macrophage cholesterol efflux and protects against atherosclerosis by augmenting the expression of ABCA1 and ABCG1. *Aging*.

[24] Zhao Z. W., Zhang M., Chen L. Y. (2018). Heat shock protein 70 accelerates atherosclerosis by downregulating the expression of ABCA1 and ABCG1 through the JNK/Elk-1 pathway. *Biochimica et biophysica acta Molecular and cell biology of lipids*.

[25] Ouimet M., Barrett T. J., Fisher E. A. (2019). HDL and reverse cholesterol transport. *Circulation research*.

[26] Chistiakov D. A., Bobryshev Y. V., Orekhov A. N. (2016). Macrophage-mediated cholesterol handling in atherosclerosis. *Journal of Cellular and Molecular Medicine*.

[27] Frambach S. J. C. M., de Haas R., Smeitink J. A. M., Rongen G. A., Russel F. G. M., Schirris T. J. J. (2019). Brothers in arms: ABCA1- and ABCG1-mediated cholesterol efflux as promising targets in cardiovascular disease treatment. *Pharmacological reviews*.

[28] Shashkin P., Dragulev B., Ley K. (2005). Macrophage differentiation to foam cells. *Current pharmaceutical design*.

[29] Ozasa H., Ayaori M., Iizuka M. (2011). Pioglitazone enhances cholesterol efflux from macrophages by increasing ABCA1/ABCG1 expressions via PPAR*γ*/LXR*α* pathway: findings from in vitro and ex vivo studies. *Atherosclerosis*.

[30] He X.-W., Yu D., Li W.-L. (2016). Anti-atherosclerotic potential of baicalin mediated by promoting cholesterol efflux from macrophages via the PPAR*γ*-LXR*α*-ABCA1/ABCG1 pathway. *Biomedicine & pharmacotherapy = Biomedecine & pharmacotherapie*.

[31] Tsai J. Y., Su K. H., Shyue S. K. (2010). EGb761 ameliorates the formation of foam cells by regulating the expression of SR-A and ABCA1: role of haem oxygenase-1. *Cardiovascular research*.

[32] Li X. Y., Kong L. X., Li J., He H. X., Zhou Y. D. (2013). Kaempferol suppresses lipid accumulation in macrophages through the downregulation of cluster of differentiation 36 and the upregulation of scavenger receptor class B type I and ATP-binding cassette transporters A1 and G1. *International journal of molecular medicine*.

[33] Gisterå A., Hansson G. K. (2017). The immunology of atherosclerosis. *Nature reviews Nephrology*.

[34] Cao X., Zhang L., Chen C. (2017). The critical role of ABCG1 and PPAR*γ*/LXR*α* signaling in TLR4 mediates inflammatory responses and lipid accumulation in vascular smooth muscle cells. *Cell and tissue research*.

[35] Hu X., Qin H., Li Y. (2020). Biochanin A protect against lipopolysaccharide-induced acute lung injury in mice by regulating TLR4/NF-*κ*B and PPAR-*γ* pathway. *Microbial pathogenesis*.

[36] Zhang Y., Chen W. A. (2015). Biochanin A inhibits lipopolysaccharide-induced inflammatory cytokines and mediators production in BV2 microglia. *Neurochemical research*.

[37] Moss J. W. E., Ramji D. P. (2016). Nutraceutical therapies for atherosclerosis. *Nature reviews Cardiology*.

[38] Garshick M., Underberg J. A. (2017). The use of primary prevention statin therapy in those predisposed to atherosclerosis. *Current atherosclerosis reports*.

[39] Mulvihill E. E., Burke A. C., Huff M. W. (2016). Citrus flavonoids as regulators of lipoprotein metabolism and atherosclerosis. *Annual review of nutrition*.

[40] Cheng Y. C., Sheen J. M., Hu W. L., Hung Y. C. (2017). Polyphenols and oxidative stress in atherosclerosis-related ischemic heart disease and stroke. *Oxidative medicine and cellular longevity*.

[41] Guo M., Lu H., Qin J. (2019). Biochanin A provides neuroprotection against cerebral ischemia/reperfusion injury by Nrf2-mediated inhibition of oxidative stress and inflammation signaling pathway in rats. *Medical science monitor : international medical journal of experimental and clinical research*.

[42] Bai Y., Li Z., Liu W., Gao D., Liu M., Zhang P. (2019). Biochanin A attenuates myocardial ischemia/reperfusion injury through the TLR4/NF-*κ*B/NLRP3 signaling pathway. *Acta cirurgica brasileira*.

[43] Geovanini G. R., Libby P. (2018). Atherosclerosis and inflammation: overview and updates. *Clinical Science*.

[44] Wolf D., Ley K. (2019). Immunity and inflammation in atherosclerosis. *Circulation research*.

[45] Xue Z., Zhang Q., Yu W. (2017). Potential lipid-lowering mechanisms of biochanin A. *Journal of Agricultural and Food Chemistry*.

[46] Ming X., Ding M., Zhai B., Xiao L., Piao T., Liu M. (2015). Biochanin A inhibits lipopolysaccharide-induced inflammation in human umbilical vein endothelial cells. *Life sciences*.

[47] Oh J. S., Cho I. A., Kang K. R. (2016). Biochanin-A antagonizes the interleukin-1*β*-induced catabolic inflammation through the modulation of NF*κ*B cellular signaling in primary rat chondrocytes. *Biochemical and biophysical research communications*.

[48] Wang D., Yang Y., Lei Y. (2019). Targeting foam cell formation in atherosclerosis: therapeutic potential of natural products. *Pharmacological reviews*.

[49] Chistiakov D. A., Melnichenko A. A., Myasoedova V. A., Grechko A. V., Orekhov A. N. (2017). Mechanisms of foam cell formation in atherosclerosis. *Journal of Molecular Medicine*.

[50] Oram J. F. (2002). Molecular basis of cholesterol homeostasis: lessons from Tangier disease and ABCA1. *Trends in molecular medicine*.

[51] Yvan-Charvet L., Ranalletta M., Wang N. (2007). Combined deficiency of ABCA1 and ABCG1 promotes foam cell accumulation and accelerates atherosclerosis in mice. *The Journal of clinical investigation*.

[52] Wang H., Yang Y., Sun X. (2018). Sonodynamic therapy-induced foam cells apoptosis activates the phagocytic PPAR*γ*-LXR*α*-ABCA1/ABCG1 pathway and promotes cholesterol efflux in advanced plaque. *Theranostics*.

[53] Yet S.-F., Layne M. D., Liu X. (2003). Absence of heme oxygenase-1 exacerbates atherosclerotic lesion formation and vascular remodeling. *FASEB journal : official publication of the Federation of American Societies for Experimental Biology*.

[54] Juan S. H., Lee T. S., Tseng K. W. (2001). Adenovirus-mediated heme oxygenase-1 gene transfer inhibits the development of atherosclerosis in apolipoprotein E-deficient mice. *Circulation*.

[55] Liu M., Yang W., Liu S. (2018). LXR*α* is expressed at higher levels in healthy people compared to atherosclerosis patients and its over-expression polarizes macrophages towards an anti-inflammatory M*Φ*2 phenotype. *Clinical and Experimental Hypertension*.

[56] Wei Z., Wang J., Shi M., Liu W., Yang Z., Fu Y. (2016). Saikosaponin a inhibits LPS-induced inflammatory response by inducing liver X receptor alpha activation in primary mouse macrophages. *Oncotarget*.

[57] Zhang M., Nakamura K., Kageyama S. (2018). Myeloid HO-1 modulates macrophage polarization and protects against ischemia-reperfusion injury. *JCI insight*.

[58] Cho R. L., Yang C. C., Tseng H. C., Hsiao L. D., Lin C. C., Yang C. M. (2018). Haem oxygenase-1 up-regulation by rosiglitazone via ROS-dependent Nrf2-antioxidant response elements axis or PPAR*γ* attenuates LPS-mediated lung inflammation. *British journal of pharmacology*.

[59] Xu W., Hu X., Qi X. (2019). Vitamin D ameliorates angiotensin II-induced human endothelial progenitor cell injury via the PPAR-*γ*/HO-1 pathway. *Journal of vascular research*.

