# Supplementary material for: Biochanin A Mitigates Atherosclerosis by Inhibiting Lipid Accumulation and Inflammatory Response
Source: Oxid Med Cell Longev. 2020 Nov 11;2020:8965047. doi: 10.1155/2020/8965047 (PMC8074550; doi:10.1155/2020/8965047)
Supplement: Supplementary Materials — Supplementary Figure 1: BCA has no effect on fat contents in the liver. Hepatic tissues were isolated from apoE−/− mice, and the levels of TC and TG were detected using commercial kits (n = 10). Data are expressed as mean ± SD. Supplementary Figure 2: evaluation of siRNA transfection efficiency. (a–c) THP-1 macrophage-derived foam cells were transfected with 50 nM of scrambled siRNA, LXRα siRNA, PPARγ siRNA, or HO-1 siRNA for 24 h, followed by Western blot assay for LXRα, PPARγ, and HO-1 expression. Data are expressed as mean ± SD from three independent experiments. ∗P < 0.05 vs. control group. Supplementary Table 1: the primer sequences used in qRT-PCR. [file 8965047.f1.zip › Supplementary Table 1.docx]

**Supplementary Table 1.** The primer sequences used in qRT-PCR.

| ABCA1 (Human) | Forward, 5’-GTCCTCTTTCCCGATTATCTGG-3’;  Reverse, 5’-CACTCACTCTCGCTCGCAAT-3’ |
| --- | --- |
| ABCA1 (Mouse) | Forward, 5’-GGGTGGTGTTCTTCCTCATTAC-3’;  Reverse, 5’-GAATGACGAGGATGAGGATGTG-3’ |
| ABCG1 (Human) | Forward, 5’-TGTTCATCAGCGTGCACTTC-3’;  Reverse, 5’-AGGGCTCAAGCATTGTCATC-3’ |
| ABCG1 (Mouse) | Forward, 5’-AGGTCTCAGCCTTCTAAAGTTCCTC-3’;  Reverse, 5’-TCTCTCGAAGTGAATGAAATTTATCG-3’ |
| SR-A (Human) | Forward, 5’-TTTGATGCTCGCTCAATGACA-3’;  Reverse, 5’-GCTGCCACTATTCCAATGAGAG-3’ |
| SR-A (Mouse) | Forward, 5’-TGGTCCACCTGGTGCTCC-3’;  Reverse, 5’-ACCTCCAGGGAAGCCAATTT-3’ |
| CD-36 (Human) | Forward, 5’-TGCTCATCTATACACGGTTACC-3’;  Reverse, 5’-TGCTCATCTATACACGGTTACC-3’ |
| CD-36 (Mouse) | Forward, 5’-CCCAGATGCACCATGGGCTTGGCAA-3’;  Reverse, ﻿5’-AAGCTCGTGCGGCCCAGGTACT-3’ |
| PPARγ (Human) | Forward, 5’-TGGAATTAGATG ACAGCGACTTGG-3’;  Reverse, 5’-CTGGAGCAGCTTGGCAAACA-3’ |
| PPARγ (Mouse) | Forward, 5’-GCAGCTACTGCATGTGATCAAGA-3’;  Reverse, 5’-GTCAGCGGGTGGGACTTTC-3’ |
| LXRα (Human) | Forward, 5’-CGATCGAGGTGATGCTTCTG-3’;  Reverse, 5’-GGCAAAGTCTTCCCGGTTAT-3’ |
| LXRα (Mouse) | Forward, 5’-AGGAGTGTCGACTTCGCAAA-3’;  Reverse, 5’-CTCTTCTTGCCGCTTCAGTTT-3’ |
| HO-1 (Human) | Forward, 5’-TTCAAGCAGCTCTACCGCTC-3’;  Reverse, 5’-GAACGCAGTCTTGGCCTCTT-3’ |
| HO-1 (Mouse) | Forward, 5’-GGTGACAGAAGAGGCTAAGA-3’;  Reverse, 5’- TCTGGCGAAGAAACTCTG-3’ |
| TNF-α (Mouse) | Forward, 5’-ACCCTCACACTCAGATCATCTT-3’;  Reverse, 5’-GGTTGTCTTTGAGATCCATGC-3’ |
| IL-1β (Mouse) | Forward, 5’-CGCAGCAGCACATCAACAAGAGC-3’;  Reverse, 5’-TGTCCTCATCCTGGAAGGTCCACG-3’ |
| IL-6 (Mouse) | Forward, 5’-TGATGGATGCTACCAAACTGGA-3’;  Reverse, 5’-TGTGACTCCAGCTTATCTCTTGG-3’ |
| β-actin (Human) | Forward, 5’-GACCTCTATGCCAACACAGT-3’;  Reverse, 5’-AGTACTTGCGCTCAGGAGGA-3’ |
| β-actin (Mouse) | Forward, 5’-TGGCACCCAGCACAATGAA-3’;  Reverse, 5’-CTAAGTCATAGTCCGCCTAGAAGCA-3’ |
