# Supplementary figures and images for: Biochanin A Mitigates Atherosclerosis by Inhibiting Lipid Accumulation and Inflammatory Response
Source: Oxid Med Cell Longev. 2020 Nov 11;2020:8965047. doi: 10.1155/2020/8965047 (PMC8074550; doi:10.1155/2020/8965047)

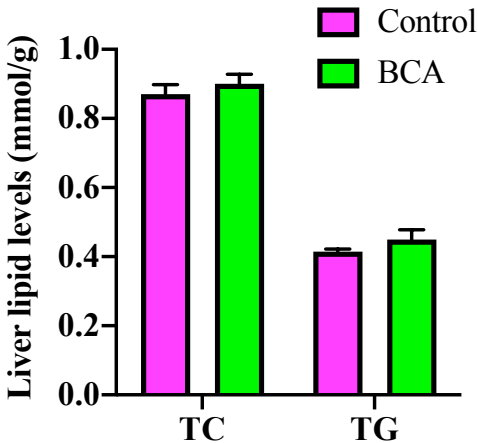

**Supplementary Figure 1**

Supplement: Supplementary Materials — Supplementary Figure 1: BCA has no effect on fat contents in the liver. Hepatic tissues were isolated from apoE−/− mice, and the levels of TC and TG were detected using commercial kits (n = 10). Data are expressed as mean ± SD. Supplementary Figure 2: evaluation of siRNA transfection efficiency. (a–c) THP-1 macrophage-derived foam cells were transfected with 50 nM of scrambled siRNA, LXRα siRNA, PPARγ siRNA, or HO-1 siRNA for 24 h, followed by Western blot assay for LXRα, PPARγ, and HO-1 expression. Data are expressed as mean ± SD from three independent experiments. ∗P < 0.05 vs. control group. Supplementary Table 1: the primer sequences used in qRT-PCR. [file 8965047.f1.zip › SF1.pdf]

**a**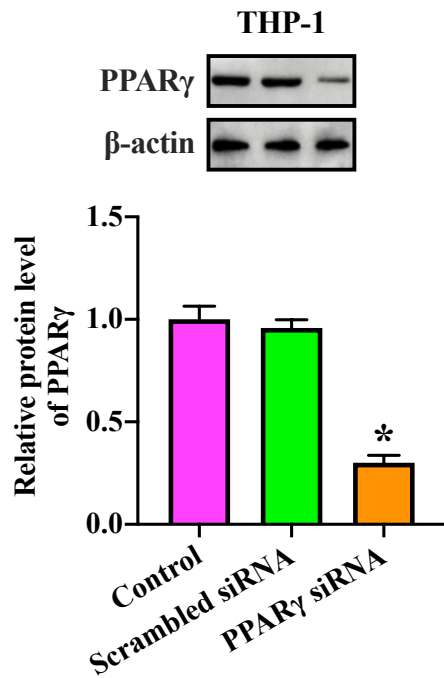**b**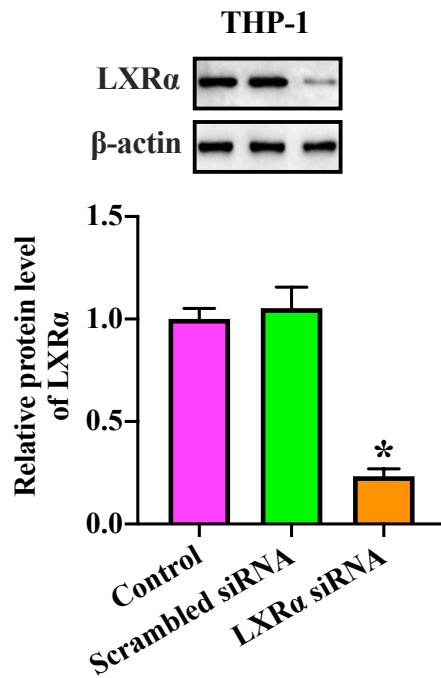**c**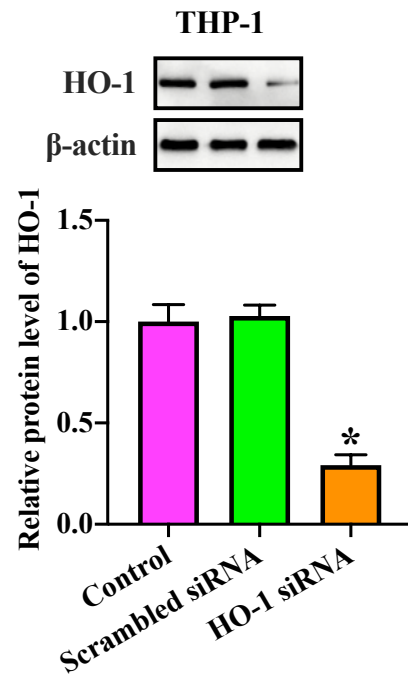**Supplementary Figure 2**

Supplement: Supplementary Materials — Supplementary Figure 1: BCA has no effect on fat contents in the liver. Hepatic tissues were isolated from apoE−/− mice, and the levels of TC and TG were detected using commercial kits (n = 10). Data are expressed as mean ± SD. Supplementary Figure 2: evaluation of siRNA transfection efficiency. (a–c) THP-1 macrophage-derived foam cells were transfected with 50 nM of scrambled siRNA, LXRα siRNA, PPARγ siRNA, or HO-1 siRNA for 24 h, followed by Western blot assay for LXRα, PPARγ, and HO-1 expression. Data are expressed as mean ± SD from three independent experiments. ∗P < 0.05 vs. control group. Supplementary Table 1: the primer sequences used in qRT-PCR. [file 8965047.f1.zip › SF2.pdf]
